# Do nurse performance appraisals reflect caring behaviors? Evidence from an Indonesian hospital

**DOI:** 10.3389/frhs.2026.1773688

**Published:** 2026-04-15

**Authors:** Kuntarti Kuntarti, Irfan Rahmayanto

**Affiliations:** 1 Basic Science and Fundamentals of Nursing Department, Faculty of Nursing, Universitas Indonesia, Depok, Indonesia; 2 Dr. Cipto Mangunkusumo Hospital, Central Jakarta, Indonesia

**Keywords:** caring behavior, caring behavior inventory (CBI-42), caring presence, health services management, individual performance index (IPI), nurse performance appraisal, patient-centered care

## Abstract

**Introduction:**

Nurses' caring behavior is central to the quality and humanistic orientation of nursing services. However, many hospital performance appraisals remain task-oriented and may insufficiently reflect caring competencies essential to patient-centered care. This study examined the association between nurses' caring behavior and Individual Performance Index (IPI) scores and identified caring dimensions requiring improvement.

**Methods:**

A cross-sectional study was conducted among 228 nurses in a public hospital in Jakarta, Indonesia, recruited through simple random sampling. Caring behavior was measured using the Caring Behavior Inventory (CBI-42), and IPI was self-entered by nurses based on their most recent official monthly appraisal report. Spearman's rank correlation was used (two-tailed, α = 0.05).

**Results:**

No significant association was found between overall caring behavior and IPI scores (*r* = 0.027; *p* = 0.680), although both measures were generally high (median CBI = 3.50 [2.00–4.00]; median IPI = 1.00 [0.80–1.10]). Positive connectedness (3.44) and attentiveness to others' experiences (3.50) were comparatively lower than the other caring dimensions.

**Discussion:**

Current appraisal systems may not adequately capture caring competencies. Integrating explicit caring indicators into nurse performance evaluation may strengthen workforce development and support patient-centered health systems.

**Conclusion:**

Nurse performance appraisal scores were not significantly associated with caring behavior, suggesting that existing appraisal systems may not adequately reflect caring competencies in nursing practice. Incorporating explicit caring-related indicators into performance evaluation may improve the alignment between assessed performance and the humanistic goals of nursing care.

## 1 Introduction

Caring behavior is a component of the appraisal of nurse performance and serves as a basis for the nursing process (1). Caring behavior is also a key concept (2) that affects nurses' performance (3) and service quality (4) and that is used for assessing their competency in terms of knowledge, skills, and clinical decision making (5). Therefore, this aspect should be included in nurse performance assessments to increase nurses’ motivation to provide quality healthcare (6).

Additionally, country- and institution-specific nurse performance appraisals should be developed based on the nursing service standards in the various countries and institutions where the nurses work. In Indonesia, nurse performance is assessed using the individual performance index (Kepmenkes Number 625/Menkes/SK/V/2010), which is generated by comparing the total results with the individual performance units for the determined and targeted components (7). The IPI components for each institution depend on their targets, although the minimum should be the same or more than those set by the government.

The IPI used at a public hospital in Jakarta is derived from a structured, monthly appraisal system which summarizes nurses' performance in terms of core performance, work behavior and supporting activities. Core performance includes the quantity and quality of nursing care provided. Quantity is evaluated based on monthly patient achievement. Quality is assessed through five indicators: application of the nursing process; implementation, including competency evaluation of selected nursing procedures; nursing documentation; application of general and specific nursing quality indicators; and customer satisfaction or complaint handling. Work behavior comprises six indicators: presence/existence, initiative, reliability, compliance, teamwork/cooperation and attitude/behavior. Supporting components include participation in scientific discussions or case conferences, serving on nursing committees, providing clinical guidance to new employees or students, completing additional tasks inside or outside the unit, and receiving consultations to deliver nursing care according to specific competencies (8).

Conceptually, caring is widely regarded as a core nursing competency and a central element of patient-centered care. Studies have associated caring behaviors with patients' sense of safety, trust and comfort, as well as improved experience and satisfaction (9–11). As the IPI is designed to reflect overall nursing performance and incorporates quality and behavioral elements, a positive correlation between caring behavior and IPI scores is plausible. However, if the operational indicators within the IPI emphasize technical/process measures, workload targets and documentation rather than interpersonal caring competencies, the relationship may be weak or absent, reflecting a misalignment of constructs between caring behavior and the performance appraisal metric.

Therefore, this study examined the relationship between nurses' caring behavior (measured by the CBI-42) and IPI scores to assess whether routine performance appraisal aligns with patient-centered caring competencies in clinical nursing practice.

## 2 Methods

### 2.1 Study designs

This research employed a quantitative cross-sectional design to analyze the correlation between caring behavior and nurse performance.

### 2.2 Setting and participants

The study was conducted in a public hospital in Jakarta, Indonesia. Inclusion criteria were nurses with at least 1 year of work experience. Exclusion criteria were nurses on leave, sick leave, or study duty during data collection. The study population consisted of 479 eligible clinical nurses. An *a priori* sample size calculation using G*Power (*r* = 0.20, *α* = 0.05, power = 0.80) indicated a minimum sample of 218. Using simple random sampling, 230 nurses were invited to account for non-response; 228 completed the survey, while two did not participate due to maternity leave.

### 2.3 Instruments

Caring behavior was measured using the Caring Behavior Inventory (CBI-42) developed by Wolf (12). The Indonesian version was translated and validated through forward–backward translation. In the present study sample (*N* = 228), the CBI-42 demonstrated excellent internal consistency (Cronbach's *α* = 0.957). The instrument assesses five dimensions: (1) Respect for others, (2) Professional knowledge and skills, (3) Positive connectedness, (4) Attentiveness to others' experiences, and (5) Attendance. Each item is rated on a 4-point Likert scale (1 = strongly disagree to 4 = strongly agree). Dimension scores and the total CBI-42 score were computed as the mean of relevant items, resulting in a possible score range of 1–4 (higher scores indicate higher caring).

The Individual Performance Index (IPI) score for each nurse in the last month was provided in a questionnaire completed by each respondent. In this hospital, the performance of individual nurses is assessed monthly using an official appraisal system that generates an Indeks Kinerja Individu (IKI) score out of 110%. This converted value is referred to as the Individual Performance Index (IPI) on a scale of 0–1.10 (IPI = IKI/100). The IKI is derived from core performance (30% quantity and 40% quality), work behavior (30%), and a supporting component (up to 10%) (8). Quantity is based on the monthly patient load, which is recorded daily and totaled at the end of the month. Targets vary according to role and unit. Quality is based on applying the nursing process (12%), competence in implementing at least two nursing procedures per month (15%), nursing documentation (5%), nursing service quality indicators (5%), and handling customer satisfaction and complaints (3%). Work behavior includes presence (15%) and initiative, reliability, compliance, teamwork and attitude/behavior (each 3%). In this study, the IPI was entered by each respondent in the questionnaire based on their most recent official monthly appraisal report immediately prior to data collection. Only the overall IPI score was collected; domain-level sub-scores were not available.

### 2.4 Data collection and ethics

Data were collected online through Google Forms between May and July 2020 (13). Each participant provided informed consent electronically. The study was approved by: the Faculty of Nursing, Universitas Indonesia (No. SK-63/UN2.F12.D1.2.1/ETIK2020), and Dr. Cipto Mangunkusumo Hospital (No. KET-342/UN2.F1/ETIK/PPM.0002/2020). All data were anonymized and stored in password-protected files.

### 2.5 Data analysis

Descriptive statistics summarized demographic data and variable distributions. The Kolmogorov–Smirnov test indicated non-normal distribution; therefore, Spearman's rank correlation was used to assess associations between CBI and IPI scores. Statistical significance was set at *p* < 0.05 (two-tailed).

## 3 Results

### 3.1 Respondent characteristics

Most respondents were women (88.2%). The median age was 31 years (22–57), and the median work period was 8.5 years (1–36) (Table 1). Education levels included Diploma/D3 (*n* = 203; 89.0%), Ners (*n* = 21; 9.2%), and SPK (*n* = 4; 1.8%), where SPK refers to a secondary nursing school qualification in Indonesia (high-school equivalent). The small SPK subgroup may reflect the relatively senior composition of the nursing workforce in this long-established national referral hospital.

**Table 1:** Respondent characteristics (*N* = 228).

Variable	Median (min–max)	*n* (%)
Age, years	31 (22–57)	
Work period, years	8.5 (1–36)	
Gender, male		27 (11.8)
Gender, female		201 (88.2)
Education, diploma		203 (89.0)
Education, Ners		21 (9.2)
Education, SPK^a^		4 (1.8)

^a^ A secondary nursing school qualification in Indonesia (high-school equivalent).

### 3.2 Nurse caring behavior

Caring behavior was measured using the CBI-42 (12); higher scores indicate better caring behavior. Because distributions were non-normal, results are presented as median (min–max). The overall CBI-42 score was 3.50 (2.00–4.00), indicating high caring behavior (Table 2). Across dimensions, the lowest median score was positive connectedness, 3.44 (2.33–4.00), followed by attentiveness to others' experiences, 3.50 (2.25–4.00). The highest median score was professional knowledge and skills, 3.80 (2.25–4.00). The attendance dimension showed a high median, 3.67 (2.17–4.00); however, the minimum observed value (2.17) indicates that a small subset of nurses reported lower caring presence.

**Table 2:** Nurses’ caring behavior in a government hospital in Jakarta (*N* = 228).

Dimension	Median (min–max)
Caring behavior	3.50 (2.00–4.00)
(1) Respect for others	3.58 (2.33–4.00)
(2) Attendance	3.67 (2.17–4.00)
(3) Positive connectedness	3.44 (2.33–4.00)
(4) Professional knowledge and skills	3.80 (2.60–4.00)
(5) Attentiveness to others' experiences	3.50 (2.25–4.00)

### 3.3 Individual performance index (IPI)

Nurse performance was assessed using the hospital's IPI appraisal system. The overall IPI score was 1.00 (0.80–1.10). Most nurses (57.0%) were classified in the “special” performance category, and no respondents were classified as below expectations (Table 3).

**Table 3:** Distribution of the individual performance Index scores of nurses working in a government hospital in Jakarta (*N* = 228).

Category (IPI's score)	*n* (%)
Excellent (>1.00)	79 (34.6)
Special (0.91–1.00)	130 (57.0)
According to expectation (0.81–0.90)	19 (8.3)
Below expectation (<0.80)	0 (0.0)

### 3.4 Relationship between nurses' caring behavior and their individual performance index scores

Spearman's rank correlation showed that the association between overall CBI-42 and IPI scores was positive but very weak (*r* = 0.027) and not statistically significant (*p* = 0.680; *α* = 0.05).

## 4 Discussion

### 4.1 Nurses' caring behavior

Using the CBI-42, nurses reported high caring behavior scores. Prior studies have noted differences in caring ratings between nurses and patients/families, with nurses sometimes rating caring higher than patients and families (14). Another study using the CBI-42 found similarly high perceptions of caring among nurses, students, and patients (15).

The respondents had the highest median score on the professional knowledge and skills subscale. This outcome showed that nurses professionally perform their duties based on competent knowledge and skills by taking action following established procedures, having confidence when caring for patients, and maintaining confidentiality with patient information (16). In addition, respondents with long working experience had good knowledge and skills. Tenure (working period) was an internal factor affecting nurse performance, such as experience, consideration, and commitment to the quality of nursing care provided (17). This result was in line with the research conducted by Respati (18) in a public hospital in Jakarta, which also obtained the highest average score, 3.5, on the caring behavior knowledge and skills subscale (ranging from 1 to 4).

Increasing the knowledge and skills of nurses is a great focus of the training of nurses in hospitals. It has been addressed through data collection, training in knowledge and skills, and examinations for increasing clinical authority. However, there has been no specific training that addresses increasing nurse caring behavior at the hospital.

The lowest median scores were observed for positive connectedness and attentiveness to others' experiences. These findings suggest that relational aspects of caring—particularly emotional connection and sensitivity to patients' experiences—remain areas for improvement (16). The positive connectedness subscale was related to carative factors, namely, physical needs, mental health, sociocultural environment support, protection, and improvement, thus applying good patient nursing care (16). A supportive physical, mental, and sociocultural environment also helped nurses apply caring behavior to perform their duties.

Despite a high median, the minimum attendance score (2.17) suggests a small subset with lower caring presence (i.e., availability and readiness to listen). As described in recent literature (19) and in Halldórsdóttir's theory, attendance reflects a caring presence, which can be life-giving and foster hope and trust (20). Conversely, absence or detachment can be life-restraining. Therefore, targeted coaching/supervision and workflow support are recommended to reduce low-end outliers and protect the patient experience.

However, all dimensions of nurses' caring behavior had a median score of more than 3.4 (on a scale of 1–4) or 80%, indicating generally high caring behavior across domain. This result was a strength for healthcare institutions because of the impact on patient satisfaction. A previous study stated a significant relationship between nurse caring behavior and patient and family satisfaction levels in the hospital (21). The quality of nursing services increased because of the good caring behavior of nurses (22).

According to Jean Watson, respect for each other subscale was related to the intervention on the ten carative factors, especially in the factors helping to fulfil human needs and appreciation for existential-phenomenological strength. The basic requirements are needed for caring behavior that has the potential to maintain the harmony of soul, mind, body, and unity in all aspects of nursing (18, 23). This subscale was related to Watson's two carative factors, namely, building relationships of mutual help and trust and increasing and accepting the expression of positive and negative feelings. A study identified that the nurses' caring behavior perceived by patients had lower values than the nurses' perceptions. Additionally, it was observed that caring behavior fostered relationships, mutual help, trust, and respect for nurses' positive and negative expressions in patients (24).

### 4.2 Executive nurse individual performance index

The average IPI score in this study was in the special performance category, and no nurses were classified as below expectations. This finding is consistent with Sulistyowati (8), which reported an average special performance index score of 0.91–1.00 with 60.3% of nurses categorized as special performance. In the present study, 57% of nurses were categorized as special performance, indicating a slight decrease that may reflect differences in respondent characteristics and assessment components.

Importantly, IPI scores showed a compressed distribution, with virtually no low performers. This restricted range likely attenuated the correlation with caring behavior; therefore, the null association should not be interpreted as definitive evidence that caring behavior is unrelated to performance.

### 4.3 Relationship between nurses' caring behavior and their individual performance index scores

The correlation between the CBI-42 and IPI median score was positive and very weak (*r* = 0.027); therefore, the relationship between nurses' caring behavior and individual performance index scores was not significant (*p* = 0.680). This outcome was caused by several factors, especially those related to the components for measuring nurses' IPIs.

The first factor was due to the one-third of the nurses' IPI score obtained from the quantity factor, namely, the number of patients treated each month. This factor was not related to caring behavior since the number of patients treated depended on the hospital's bed occupancy ratio (BOR) (8). The percentage of beds used in a certain unit was then used as a reference in the nursing field to carry out the assignment method and control the nurses’ range. Therefore, each nurse's target achievement was observed every month. The component weight of 30% was sufficient to influence each nurse's final IPI score.

The second factor influencing these results was the nurses' caring behavior, which was included in Watson's carative factors; however, this factor was not explicitly written as a component in the nurse's performance assessment, which produces the IPI score. The second component produced the nurses' main performance index, namely, the nursing process application and implementation (competency evaluation of at least two nursing actions per month), documentation, application of general quality indicators and specific services, and customer satisfaction or the handling of complaints. This outcome occurred because the above components do not explicitly mention screening as the component assessed in the IPI.

In addition to the two components above, as employees work for public agencies, nurses were also assessed on their work behavior, including existence, initiative, reliability, compliance, cooperation, and attitude-behavior. Nurses' caring behavior shows a professional attitude that reflects a desire to increase knowledge, initiative, skills, and reliability (25). One of the five caring processes was physical contact, which refers to being in attendance or presence/existence. Therefore, nurses should be attentive and express readiness to listen to the patient's problems or complaints. This act fosters solutions to patients' health problems (26).

Another assessment component was supporting. This dimension included the nurses' participating in scientific discussions/conferences, being on the committee for nursing activities, providing frequent clinical guidance to new employees/students, carrying out additional tasks inside and outside the work unit, or receiving consultations in providing care to patients according to their specific competencies. Although caring was the basis of implementing the above component, there was a need for caring behavior to be reflected in the IPI assessment for the executive nurse.

The third factor was subjectivity, which distinguishes the assessment results of the head nurses from each ward. The head nurse's individual performance index assessment was carried out at the assigned place. Therefore, each head nurse's policies in conducting the assessment were different. The final IPI results from each room were thus different, even with the same agreed-upon measuring instrument. One study stated that one of the problems of the nurse performance appraisal system lies in assessment subjectivity (27).

A plausible explanation for the non-significant association is construct misalignment between the caring construct and the operational indicators within the IKI/IPI. While the IPI includes quality and behavioral components, a substantial portion of the score reflects technical/process indicators (e.g., applying the nursing process, procedural competence, documentation) and quantity targets. These indicators may not fully capture interpersonal caring behaviors (e.g., empathy, connectedness, attentiveness) measured by the CBI-42, which could attenuate the observed relationship.

### 4.4 Limitations of the IPI as a performance tool

The limitations encountered included the different perceptions among nurses and taking the sample in one place. Ideally, the nurses' caring behavior should be compared with patient perceptions, and both produce good values. The sampling was carried out to examine the two relationships between the caring behavior variable and executive nurses' IPI scores. Therefore, it is necessary to sample additional hospitals and institutions to determine the correlation between the two research variables and compare the results obtained from each hospital. Performance appraisal reform is necessary to incorporate explicit caring dimensions—such as communication, empathy, and patient satisfaction—to ensure holistic evaluation consistent with the goals of patient-centered health systems.

### 4.5 Implications for patient-centered health systems

This research is recommended as an input for hospitals, as it explains that the IPI value should also reflect nurses' caring behavior. Integrating caring indicators into performance appraisal systems could transform how institutions value compassionate care. A patient-centered system prioritizes not only efficiency but also human connection and emotional safety. Hospitals could consider: (1) including caring behavior items in IP rubrics; (2) linking appraisal outcomes with patient satisfactory feedback; (3) providing recognition programs for exemplary caring behaviors; and (4) offering reflective training on empathy and positive connectedness.

Several caring behavioral dimensions had moderate scores. Even though these scores were still good, the results suggested the need for nurses to continue to improve. Additionally, the results of this study may serve as input on the effectiveness of performance appraisals for nurses in health services and serve as a reference for further study to support the development of an improved performance appraisal instrument for use in the nursing profession.

### 4.6 Limitations and future research

As this study was conducted in a single institution, the findings may not be generalisable to other healthcare settings. Caring behavior was assessed using self-report by nurses, which may be influenced by social desirability and may not fully reflect patients' experiences. Future research should include multiple hospitals and use multiple methods to assess caring behavior, such as patient-reported measures of caring or patient experience, alongside nurse ratings. Furthermore, longitudinal or prospective designs are recommended to explore potential causal pathways between caring behavior, performance ratings and patient outcomes.

## 5 Conclusion

This study found no significant association between nurses' caring behavior (as measured by the CBI-42) and IPI scores, although both measures were generally high. Professional knowledge and skills achieved the highest scores for caring, while positive connectedness and attentiveness to others' experiences achieved comparatively lower scores. This indicates priority areas for strengthening the relational aspects of patient-centered care. IPI scores were concentrated in the higher categories, with no nurses performing below expectations. This suggests restricted variability, which may limit the appraisal system's ability to differentiate performance and capture caring competencies.

These findings suggest that hospitals should not rely solely on IPI to recognize or incentivize caring behavior. Instead, performance evaluation frameworks should incorporate explicit caring indicators (e.g., communication, empathy, attentiveness and a caring presence) and, where feasible, integrate patient-reported caring or patient experience measures. Clearer scoring rubrics and rater calibration are recommended to improve appraisal objectivity and alignment with patient-centered care goals.

## Data Availability

The datasets generated and analyzed during the current study are available from the corresponding author on reasonable request.
