# Correction: Do nurse performance appraisals reflect caring behaviors? Evidence from an Indonesian hospital

**DOI:** 10.3389/frhs.2026.1857044

**Published:** 2026-05-29

**Authors:** Kuntarti Kuntarti, Irfan Rahmayanto

**Affiliations:** 1 Basic Science and Fundamentals of Nursing Department, Faculty of Nursing, Universitas Indonesia, Depok, Indonesia; 2 Dr. Cipto Mangunkusumo Hospital, Central Jakarta, Indonesia

**Keywords:** caring behavior, caring behavior inventory (CBI-42), caring presence, health services management, individual performance index (IPI), nurse performance appraisal, patient-centered care

Text correction

Adding/removing text

An error in terminology was identified in the published article, where the term “curative factors” was used instead of the correct term “carative factors,” as defined in the Theory of Human Caring by Jean Watson. The term “carative” originates from the concept of “caring,” which is a distinctive philosophical foundation of nursing science, and is intentionally differentiated from “curing,” a concept more closely aligned with the medical model. Therefore, this correction is important to preserve theoretical accuracy and prevent potential conceptual misunderstanding among readers.

A correction has been made to the section [Results, Paragraph 2]:

“[The subscale was related to carative factors]”

A correction has been made to the section Discussion, Paragraph 5 (before subsection 4.2):

“This subscale was related to Watson’s two carative factors..”

A correction has been made to the section Discussion, Subsection 4.3, Paragraph 3:

“which was included in Watson’s carative factors”

